# Impact of Adverse Socioeconomic Conditions on Physical and Oral Health Limitations Among American Older Adults

**DOI:** 10.7759/cureus.94363

**Published:** 2025-10-11

**Authors:** Alexa Lombardi, Fatimah Alobaidi, Wael Sabbah

**Affiliations:** 1 Faculty of Dentistry and Oral and Craniofacial Sciences, King’s College London, London, GBR

**Keywords:** adults, health inequalities, health status, oral health, socioeconomic factors

## Abstract

Aim

This study aims to investigate whether income and education are associated with physical, oral, and combined functional limitations among Americans aged 60 and older.

Methods

Data from the 2021-2023 National Health and Nutrition Examination Survey (NHANES) were used. Ethical approval was not required for this analysis. Physical and oral health limitations were assessed using items from the physical functioning and oral health impact questionnaires, respectively. Two binary physical and oral limitation variables were created. An additional binary combined limitation variable was also created to reflect the presence of at least one physical or oral limitation. Socioeconomic factors were indicated by income and education. Negative binomial regression was used to assess associations between socioeconomic indicators and each outcome, adjusting for age, gender, race/ethnicity, marital status, health insurance, and smoking status. NHANES sampling weights were applied to account for the complex survey design.

Results

The analysis included 2888 participants aged 60 and older. Physical, oral, and combined functional limitations were reported by 81.7% (n = 2360) (95% confidence interval {CI}: 79.0, 84.1), 66.9% (n = 1932) (95% CI: 64.0, 69.7), and 90.0% (n = 2599) (95% CI: 88.2, 91.6) of the participants, respectively. Socioeconomic disadvantage was associated with a higher risk of all types of functional limitation. Compared to the participants in the highest-income group, those in the lowest group had a greater risk of physical limitation (rate ratio {RR} = 1.35; 95% CI: 1.21, 1.51), oral limitation (RR = 1.60; 95% CI: 1.35, 1.90), and combined limitation (RR = 1.46; 95% CI: 1.29, 1.65). Similarly, those with a high school education or more had higher risks across all outcomes compared to those with a college education or more.

Conclusion

The analysis demonstrated greater inequality in physical limitations when combined with oral health limitations. There was a clear income and education gradient in physical, oral, and combined functional limitations among older adults. The income gradient was steeper for combined limitations than for physical limitations alone. Identifying those subjected to adverse economic conditions could help target vulnerable populations and inform public health interventions for oral and general health.

## Introduction

Maintaining physical and oral function is crucial for health, well-being, and independent living in older age [1,2]. As the number of older adults in the United States rises [3], an increasing number of individuals are experiencing impairments that make it harder for them to perform daily tasks or maintain their oral health [4,5]. In addition to being highly prevalent, these limitations have a major negative impact on the quality of life by lowering autonomy, increasing dependency, and raising healthcare needs [6]. The loss of independence and an increased risk of institutionalization may arise from physical functional limitations, such as difficulties with activities of daily living (ADLs), including walking, climbing stairs, lifting objects, or completing self-care chores [7,8]. Pain, trouble eating, and embarrassment regarding the mouth are examples of oral health limitations that can affect one’s physical and mental health, decrease social contact, and lead to nutritional deficiencies [9,10].

Physical and oral health limitations typically occur concurrently, especially in older adults, even though they are frequently studied independently [11]. These coexisting conditions may indicate broader patterns of disadvantage that build up over the course of a person’s life. Compared to people who only have oral or physical limitations, people who have both kinds of limitations may have worse general health, less access to quality care, and more trouble managing daily life [11-13]. More integrated approaches that consider the connection between oral and physical health are necessary, according to recent data, especially for socially vulnerable groups [7].

Socioeconomic factors, particularly income and level of education, are important determinants of oral health and physical functioning, according to previous evidence [7,14]. Throughout life, poor oral health outcomes, chronic disease, and disability are linked to lower income and education levels [9]. Health literacy, exposure to cumulative disadvantage, preventative behaviors, and healthcare access are all influenced by these socioeconomic determinants [15]. While income has a direct impact on access to services and health-related resources [16], educational attainment may affect a person’s capacity to comprehend and act upon health information [17].

Access to healthcare services is also largely dependent on having health insurance in the United States. Although insurance status is not typically thought of as a direct socioeconomic indicator, it does represent a person’s capacity to interact with and gain from the healthcare system. The lack of insurance or insufficient coverage may further limit access to care for older adults, many of whom have complicated medical and dental needs, and therefore exacerbate existing health inequities [18,19]. A more thorough knowledge of the obstacles older adults, particularly those in lower socioeconomic groups, confront can be gained by incorporating insurance status into assessments of functional health.

Few studies have concurrently examined physical and oral health limitations in this context [7,8,20,21]. Despite the well-established significance of socioeconomic status in influencing health outcomes, most of the current research looks at these areas separately, which could understate the overall burden of functional restrictions on older adults. Furthermore, it is also uncertain if considering both domains at once makes the socioeconomic gradient more noticeable. It may indicate the existence of cumulative disadvantage and assist in identifying older adults who are more at risk if combined limitations exhibit a stronger pattern by poverty or education than either physical or oral limitations alone. By investigating socioeconomic disparities in older American individuals’ physical, oral, and combined functional limitations, this study fills these gaps. The study aims to examine the associations between socioeconomic conditions, specifically income and education, and physical, oral, and combined functional limitations among older adults aged 60 years and older in the United States. In addition, the study investigates whether these associations persist after adjusting for key demographic and behavioral factors.

## Materials and methods

Study design and sample

This study was based on a secondary data analysis of the National Health and Nutrition Examination Survey (NHANES) from 2021 to 2023. The NHANES is a large, ongoing study run by the US Centers for Disease Control and Prevention (CDC). NHANES has a multistage, stratified probability sampling approach, which accurately represents the independent US population. The survey is conducted every two years and collects data through home interviews and mobile examination centers. A total of 11933 participants were assessed in NHANES, collecting data on demographics, diet, health history, physical examination results, and laboratory data to give a comprehensive health profile of the participants. The inclusion criteria for this study included participants aged 60 or older and who had complete data on socioeconomic factors, oral health and physical functioning, and covariates. Further methodological details are available from the National Center for Health Statistics [22]. The NHANES program received approval from the Ethics Review Board, and all participants gave written informed consent; details are available at https://www.cdc.gov/nchs/nhanes/about/erb.html.

Data collection

Outcome Variables

Three outcome variables were employed. Physical limitation was assessed using eight items from the NHANES physical functioning questionnaire. The participants were asked these questions, focusing on issues with walking, ascending stairs, lifting things, manual dexterity, dressing, communicating, focusing, self-care, seeing, and hearing, with responses coded as “no difficulty,” “some difficulty,” “much difficulty,” or “unable to do.” Binary values were then created (0 = no difficulty and 1 = any difficulty, including “some difficulty,” “a lot of difficulty,” or “cannot do”). The total of these eight items was used to produce a single impact variable on physical functional limitation [23]. Higher scores indicated greater physical limitations, and the total score varied from 0 to 8.

Oral health functional limitation was assessed using six items from the NHANES oral health questionnaire. These items asked participants if they experienced aching, felt bad, avoided foods, had difficulty eating, were embarrassed, or had trouble at work because of oral health problems, with responses as “never,” “rarely,” “sometimes,” “often,” and “always.” To create a single impact variable on oral health limitation, these factors were summed together and categorized as 0 (“never or rarely”) or 1 (“sometimes” to “always”) [24]. Scores were summed (range: 0-6), with higher values denoting greater oral constraints. Responses such as “refused,” “don’t know,” or “missing” were categorized as missing for both oral and physical functional limitations.

The “combined limitation” variable was created to show if a participant had at least one limitation in either the oral or physical health domains (score of ≥1 on either scale = 1 and otherwise = 0). Those with missing values for both components were identified as missing.

Exposure Variables

Socioeconomic status was measured using two indicators. Educational attainment was categorized into three categories: (1) high school diploma or more, (2) some college or associate degree, and (3) college degree or more. NHANES calculated the poverty-income ratio (PIR), which is the ratio of household income to the federal poverty threshold. The participants were divided into quartiles ranging from lowest to highest income, with the highest-income group serving as the reference category [25].

Covariates

Several behavioral and demographic factors were considered by the models. Age was included as a continuous variable, and gender was coded as male or female. Race and ethnicity were grouped into the following four categories: non-Hispanic White (reference group), non-Hispanic Black, Hispanic, and other or mixed race. Smoking status was determined based on whether the participants had ever smoked at least 100 cigarettes and whether they currently smoked. “Never smokers” were defined as those who had never smoked. Individuals who reported now smoking were labelled “current smokers,” while those who had smoked at least 100 cigarettes but did not currently smoke were labelled “former smokers.” Marital status was divided into two categories: unmarried and married or cohabiting (reference group). The participants’ self-reported insurance status was used to classify health insurance coverage as “yes” or “no.”

Statistical analysis

All analyses were conducted using Stata (StataCorp LLC, College Station, TX). Descriptive statistics were first computed to summarize participant characteristics and the distribution of functional limitations across demographic and socioeconomic groups. Means and weighted percentages with 95% confidence intervals (CIs) were reported. Negative binomial regression was then used to examine the association between socioeconomic status and each of the three outcome variables. Model 1 estimated rate ratios (RRs) for physical functional limitation, Model 2 for oral health limitation, and Model 3 for combined limitation. The primary exposures were education and poverty-income quartiles, and the models were adjusted for age, gender, race/ethnicity, smoking status, marital status, and health insurance. Results are presented as adjusted rate ratios (RRs) with 95% CIs. Stata survey commands with NHANES-provided examination sample weights were applied throughout the analysis to ensure national representativeness and to account for oversampling, stratification, and clustering.

## Results

The final analysis included 2888 participants who were 60 years of age or older and who had comprehensive data on confounders, socioeconomic indicators, dental health, and physical functioning.

The sample’s weighted mean age was 69.5 years. Men made up 46.4% of the sample, while women made up a small majority (53.6%). Many participants (72.6%) identified as non-Hispanic White, followed by Black (8.5%), Hispanic (9.6%), and other or mixed race (9.3%). In terms of educational achievement, 38.9% had only completed high school, 28.3% had some college or an associate degree, and 32.8% had completed college or more. With 27.2% of households in the highest-income group and 21.1% in the lowest, household income was spread equally according to poverty-income ratio quartiles. Current smokers included 12.1% of the sample, 35.1% had previously smoked, and more than half (52.9%) had never smoked. Most individuals (97.5%) indicated that they had health insurance. Finally, 39.8% were single, while the majority (60.2%) were married or living together. In terms of functional limitation, oral health limitations were reported by 66.93% of the participants, whereas physical functional limitations were recorded by 81.68%, and 90.03% of the participants had the combined limitation variable, which indicates the existence of at least one oral or physical constraint (Table 1).

**Table 1:** Distribution of all variables included in the analysis among 2888 participants in the NHANES 2021-2023 dataset *P ≤ 0.05 **P ≤ 0.01 ***P ≤ 0.001 from chi-squared/t-test NHANES, National Health and Nutrition Examination Survey; CI, confidence interval

Variable		Unweighted count (%), mean (95% CI)	Physical limitation (95% CI)	Oral limitation (95% CI)	Combined limitation (95% CI)
Gender	Male	1339, 46.36 (44.88, 47.84)	2276, 78.84* (75.88, 81.53)	1994, 69.05 (66.11, 71.84)	2571, 89.05 (86.54, 91.14)
	Female	1549, 53.64 (52.16, 55.12)	2430, 84.14* (80.48, 87.22)	1881, 65.1 (60.84, 69.12)	2624, 90.89 (88.06, 93.1)
Mean age		69.49 (68.95, 70.03)	69.897 (69.33, 70.47)	69.46 (68.90, 70.02)	69.65 (69.09, 70.22)
Race/ethnicity	White	2095, 72.55 (67.81, 76.84)	2381, 82.5 (79.08, 85.46)	1872, 64.81** (61.73, 67.76)	2590, 89.7* (87.23, 91.73)
	Hispanic	277, 9.582 (5.852, 15.3)	2239, 77.49 (72.16, 82.05)	2035, 70.46** (62.8, 77.12)	2550, 88.28* (82.07, 92.53)
	Black	247, 8.541 (6.139, 11.77)	2414, 83.6 (72.35, 90.85)	2109, 73.03** (68.18, 77.39)	2681, 92.83* (87.34, 96.05)
	Mixed race/other	269, 9.326 (7.608, 11.39)	2248, 77.91 (72.3, 82.66)	2144, 74.22** (69.05, 78.79)	2654, 91.91* (86.78, 95.16)
Education	High school or more	1123, 38.88 (33.96, 44.03)	2489, 86.22** (82.34, 89.37)	2078, 71.92*** (67.05, 76.32)	2692, 93.24** (90.12, 95.43)
	Some college	817, 28.31 (25.55, 31.25)	2356, 81.55** (77.56, 84.97)	1987, 68.77*** (63.67, 73.44)	2619, 90.68** (86.47, 93.67)
	College or more	948, 32.81 (27.02, 39.17)	2206, 76.41** (71.82, 80.46)	1716, 59.43*** (55.73, 63.02)	2474, 85.68** (81.85, 88.82)
Poverty-income	Lowest income	609, 21.1 (17.62, 25.06)	2526, 87.44*** (83.31, 90.66)	2225, 77.13*** (73.43, 80.46)	2715, 94.01*** (90.91, 96.09)
	Second-lowest income	703, 24.33 (21.28, 27.67)	2467, 85.48*** (81.5, 88.72)	2038, 70.6*** (66.14, 74.7)	2671, 92.5*** (89.19, 94.85)
	Second-highest income	790 27.34 (23.78, 31.2)	2335 80.89*** (77.2, 84.11)	1946 67.41*** (63.69, 70.94)	2642 91.49*** (89.6, 93.06)
	Highest income	786, 27.23 (22.07, 33.08)	2155, 74.63*** (71.19, 77.78)	1596, 55.25*** (49.54, 60.82)	2405, 83.29*** (79.71, 86.35)
Smoking status	Never smoked	1528, 52.89 (49.67, 56.08)	2295, 79.44* (76.06, 82.46)	1809, 62.66*** (59.09, 66.1)	2521, 87.24*** (84.81, 89.32)
	Former smoker	1013, 35.06 (32.77, 37.43)	2437, 84.37* (81.29, 87.02)	2013, 69.69*** (66.83, 72.4)	2675, 92.65*** (89.98, 94.65)
	Current smoker	348, 12.05 (10.07, 14.36)	2417, 83.7* (76.36, 89.09)	2242, 77.65*** (73.41, 81.38)	2734, 94.7*** (90.42, 97.13)
Health insurance	No	74, 2.55 (1.742, 3.719)	2211, 76.58 (66.96, 84.06)	2178, 75.45 (64.68, 83.77)	2628, 91.03 (84.63, 94.93)
	Yes	2814, 97.45 (96.28, 98.26)	2363, 81.82 (79.07, 84.28)	1926, 66.7 (63.74, 69.54)	2599, 90.01 (88.06, 91.67)
Marital status	Married/cohabiting	1737, 60.17 (56.62, 63.63)	2256, 78.12** (74.23, 81.57)	1835, 63.52** (59.88, 67.02)	2541, 88.00** (85.05, 90.43)
	Unmarried	1151, 39.83 (36.37, 43.38)	2514, 87.06** (83.77, 89.77)	2081, 72.07** (68.31, 75.55)	2689, 93.11** (90.78, 94.88)

Model 1 of the negative binomial regression, which examined physical functional limitations, showed that lower socioeconomic status was strongly associated with a higher risk of limitation. The participants with only a high school education (RR = 1.31; 95% CI: 1.19, 1.44) or some college education (RR = 1.21; 95% CI: 1.09, 1.35) showed higher risks compared to those with a college degree or higher. A clear income gradient was also evident, with individuals in the lowest-income quartile (RR = 1.35; 95% CI: 1.21, 1.51) and the second-lowest quartile (RR = 1.27; 95% CI: 1.16, 1.38) showing a greater likelihood of physical limitation (Table 2).

**Table 2:** Negative binomial regression showing rate ratio (RR) for factors associated with physical, oral, and combined functional limitations among older American adults (60+) (NHANES 2021-2023) (n = 2888) *P ≤ 0.05 **P ≤ 0.01 ***P ≤ 0.001 NHANES, National Health and Nutrition Examination Survey; CI, confidence interval

Negative binomial regression		Model 1 physical limitation, RR (95% CI)	Model 2 oral limitation, RR (95% CI)	Model 3 combined limitation, RR (95% CI)
Gender (reference: male)	Female	1.02 (0.94, 1.12)	0.91* (0.82, 1.00)	0.97 (0.90, 1.05)
Mean age		1.02*** (1.01, 1.03)	1.00 (0.99, 1.00)	1.01*** (1.01, 1.02)
Race/ethnicity (reference: White)	Hispanic	1.01 (0.88, 1.15)	1.10 (0.97, 1.26)	1.05 (0.93, 1.18)
	Black	0.93 (0.78, 1.10)	1.13 (0.97, 1.31)	1.02 (0.91, 1.13)
	Mixed race/other	0.92 (0.83, 1.01)	1.12** (1.04, 1.21)	1.00 (0.94, 1.08)
Education (reference: college or more)	High school or more	1.31*** (1.19, 1.44)	1.19** (1.08, 1.32)	1.26*** (1.15, 1.37)
	Some college	1.21*** (1.09, 1.35)	1.11 (0.99, 1.24)	1.17** (1.06, 1.28)
Poverty-income rate (reference: highest income)	Lowest income	1.35*** (1.21, 1.51)	1.60*** (1.35, 1.90)	1.46*** (1.29, 1.65)
	Second-lowest income	1.27*** (1.16, 1.38)	1.49*** (1.24, 1.79)	1.36*** (1.23, 1.51)
	Second-highest income	1.19*** (1.09, 1.32)	1.27** (1.08, 1.48)	1.23*** (1.12, 1.35)
Smoking status (reference: never smoked)	Former smoker	1.10* (1.018, 1.18)	1.15** (1.06, 1.25)	1.12*** (1.05, 1.19)
	Current smoker	1.14*** (1.08, 1.21)	1.27*** (1.16, 1.40)	1.20*** (1.13, 1.27)
Health insurance (reference: yes)	No	1.15** (1.05, 1.25)	1.18 (0.94, 1.47)	1.01 (0.84, 1.22)
Marital status (reference: married/cohabiting)	Unmarried	1.15** (1.05, 1.25)	1.13* (1.00, 1.27)	1.14** (91.04, 1.24)

Model 2, which assessed oral health functional limitations, revealed a similar pattern. The participants with a high school education or less (RR = 1.19; 95% CI: 1.08, 1.32) were significantly more likely to experience oral limitations compared to those with higher education. Income demonstrated a strong gradient, with those in the lowest-income (RR = 1.60; 95% CI: 1.35, 1.90), second-lowest (RR = 1.49; 95% CI: 1.24, 1.79), and second-highest quartiles (RR = 1.27; 95% CI: 1.08, 1.48) being at higher risk.

In Model 3, which examined combined physical and oral limitations, the socioeconomic gradient became even steeper. The participants in the lowest-income quartile (RR = 1.46; 95% CI: 1.29, 1.65) and those with a high school education (RR = 1.26; 95% CI: 1.15, 1.37) were substantially more likely to report combined limitations compared to those with higher education and income.

Behavioral and demographic characteristics were also associated with functional health outcomes across all models. Current smokers (RR = 1.20; 95% CI: 1.13, 1.27) and former smokers (RR = 1.12; 95% CI: 1.05, 1.19) had greater risks of functional limitations than never smokers. Unmarried participants showed higher risks of physical (RR = 1.15; 95% CI: 1.05, 1.25) and combined limitations (RR = 1.14; 95% CI: 1.04, 1.24). Although health insurance status was associated with physical limitations, it did not significantly affect oral or combined outcomes.

## Discussion

The purpose of this study was to investigate how older American adults’ socioeconomic status related to their combined oral and physical functional limitations. Using nationally representative data, the study found that lower income and education levels were significantly associated with greater physical, oral, and combined functional limitations in older adults. Considering both oral and physical health revealed stronger socioeconomic gradients, suggesting the value of a combined approach in identifying inequality.

Life course theories, which propose that cumulative exposure to socioeconomic disadvantage results in worsening health outcomes in later life, are consistent with the observed gradients [26,27]. Prior research has similarly shown that poorer physical functioning and greater difficulties with oral health are linked to lower income and education levels [8,28]. Our results expand that comparing physical and oral health metrics together can highlight more pronounced differences. Although few studies have used multidimensional techniques to measure functional health in older individuals in the United States, this aligns with recent calls to do so [9].

Although previous studies have often examined oral and physical limitations independently, this analysis demonstrates that combined limitations may more accurately reflect the compounding effects of social disadvantage. These results are consistent with earlier research by Rahmati et al., who found that one of the main factors influencing older adults’ physical functioning was social disparity [8]. Similarly, prior research has highlighted how socioeconomic hardship and systemic racism shape functional outcomes, especially in marginalized populations [7]. The findings extend these ideas to dental health, an area often overlooked in aging research, and demonstrate that oral restriction not only is widespread but also may be more sensitive to income-related disparity than physical limitation alone, which is the focus of much of this literature.

Inequalities in oral health in the United States are well-documented and have been associated with the lack of insurance coverage, high treatment costs, and unequal access to dental care [9,10]. Public insurance options for dental care for older adults exacerbate these inequities. Dental coverage is often omitted or severely restricted, especially under Medicare, even though most participants in this sample reported having health insurance. This may explain why health insurance status was not linked to oral or combined outcomes, despite being associated with physical limitations, and why oral health limitations were more strongly correlated with wealth. It also supports the findings of Knorst et al. who highlighted the important impact that structural barriers, such as cost, coverage gaps, and geographic access, play in determining older adults’ dental health [9].

These results also address a gap in aging, as oral health is frequently treated as secondary or excluded from the assessments of function. However, our findings suggest that incorporating oral health could be crucial in determining which patients require the most extensive care. Oral health constraints such as discomfort or embarrassment can directly affect nutrition, social engagement, and mental health [29]. The need for considering oral and physical function as interrelated facets of health is further underscored by their frequent co-occurrence with physical disabilities, which may indicate a deeper accumulation of disadvantage.

Complex interactions between historical disadvantage, discrimination, and structural inequalities often shape ethnic differences in functional outcomes. According to Nazroo et al., racialized disadvantage, which includes structural obstacles to healthcare, education, and income, must be considered when examining racial disparities in health in the United States [30]. The increased risk of oral restriction among the participants who identified as mixed race or “other” underscores the need for more detailed and culturally sensitive research in future work, even if this study did not find consistent trends by race and ethnicity after statistical adjustment. Furthermore, controlling for socioeconomic status may obscure the full magnitude of ethnic health disparities if racial minorities experience structural disadvantage. Following Nazroo et al., future research should investigate racism as a primary source of cumulative disadvantage in health, rather than merely as a background condition [30].

Poorer functional outcomes were also associated with other social and behavioral characteristics, including marital status and smoking. These results are aligned with previous research showing that smoking raises the risk of inflammation, oral pathology, and chronic illness [7]. The long-term effects of tobacco exposure are evident in higher risks in ex-smokers. Research also shows that living alone can reduce social support, increase stress, and limit access to informal care, all of which have an impact on oral and physical function. This is consistent with the higher limitations among single people [8]. Taken together, these findings imply that any comprehensive model of aging and functional health needs to take behavioral and social environment into account.

This study has several strengths. It employs robust survey-weighted regression techniques to account for important confounders and uses recent data from the nationally representative NHANES survey. The introduction of a combined functional limitation variable, which provides a more nuanced understanding of the interactions between socioeconomic disadvantage and other health issues, is another strength. The argument for including dental factors in public health models of aging is strengthened by treating oral health as a fundamental functional metric. Furthermore, by considering how socioeconomic determinants compound and overlap to impact health outcomes, our approach supports expanding efforts to understand health inequities through a multidimensional and intersectional lens. There are, however, several limitations. First, the cross-sectional design makes it more difficult to establish causal links between functional limitations and socioeconomic determinants. To evaluate directionality and temporal sequences, longitudinal data would be required. Second, functional and oral health limitations were assessed solely through self-reported questionnaires. This approach may introduce recall bias, subjective variation, or social desirability bias, particularly for sensitive issues such as dental health. Moreover, no objective functional assessments (e.g., grip strength, clinical dental examinations, or performance-based tests) were available, which could have provided a more accurate evaluation of the participants’ functional status. Third, the study’s ability to identify significant differences in certain racial or ethnic subgroups may have been limited by small sample sizes. Lastly, although this study concentrated on wealth and education, it did not evaluate other types of social inequality that can possibly influence differences in functioning, such as food poverty, housing instability, or the burden of caregiving. Finally, while chronic diseases such as diabetes, arthritis, depression, or cognitive impairment are known to contribute to functional limitations, these were not adjusted for in the present analyses to maintain the study’s specific focus. This should be considered when interpreting the findings, and future research could explore the interplay between chronic conditions, socioeconomic determinants, and functional outcomes.

The results suggest several important research and policy considerations. Enhancing access to dental and medical care for socioeconomically disadvantaged older adults remains a key public health priority. Policy changes that expand coverage and reduce barriers to care are particularly relevant, as public insurance programs such as Medicare often provide limited oral health services. Integrated medical-dental care models, community outreach, and preventive services may help address unmet needs. Public health initiatives targeting aging populations should also consider behavioral health, housing, and social relationships as influential factors. Future research should employ longitudinal designs to explore how intersectional disadvantages, life course exposure to adversity, and structural inequities affect functional aging across diverse racial and ethnic groups.

More broadly, this study shows that including oral health limitations alongside physical limitations provides a more comprehensive view of socioeconomic disparities in functional health among older US adults. The combined outcome measure highlights the compounded disadvantage experienced by those with low income and education. Addressing these gaps will require systemic improvements in access to both dental and medical care, as well as equity-focused, integrated public health strategies. Efforts to promote healthy aging should target the broader social determinants of functional health throughout the life course, in addition to providing clinical care.

## Conclusions

This study showed that physical limitations tend to be more pronounced when considered alongside oral health. Physical, oral, and combined functional impairments among older adults were strongly associated with income and education levels. The steeper gradient observed for combined limitations suggests that financial disadvantage is linked to greater overall functional burden. However, due to the cross-sectional design and reliance on self-reported measures, these findings reflect associations rather than causal relationships and should be interpreted with caution. Future research, particularly longitudinal studies, is needed to clarify these relationships. Nevertheless, the results highlight the importance of integrated public health programs that consider both oral and general health, with particular attention to older adults experiencing socioeconomic disadvantage.
